# Verbesserte Qualität gelagerter Erythrozytenkonzentrate durch maschinelle Autotransfusion

**DOI:** 10.1007/s00101-022-01189-6

**Published:** 2022-08-15

**Authors:** F. Münch, A. Purbojo, F. Wenzel, M. Kohl, S. Dittrich, M. Rauh, R. Zimmermann, N. Kwapil

**Affiliations:** 1 Kinderherzchirurgische Abteilung, Universitätsklinikum Erlangen, Friedrich-Alexander-Universität Erlangen-Nürnberg (FAU), Loschgestraße 15, 91054 Erlangen, Deutschland; 2 Fakultät Medical and Life Science, Hochschule Furtwangen University Campus Villingen-Schwenningen, Jakob-Kienzle-Str. 17, 78054 Villingen-Schwenningen, Deutschland; 3 Kinderkardiologische Abteilung, Universitätsklinikum Erlangen, Friedrich-Alexander-Universität Erlangen-Nürnberg (FAU), Loschgestraße 15, 91054 Erlangen, Deutschland; 4 Klinisches Labor der Kinder- und Jugendklinik, Universitätsklinikum Erlangen, Friedrich-Alexander-Universität Erlangen-Nürnberg (FAU), Loschgestraße 15, 91054 Erlangen, Deutschland; 5 Transfusionsmedizinische und Hämostaseologische Abteilung, Universitätsklinikum Erlangen, Friedrich-Alexander-Universität Erlangen-Nürnberg (FAU), Krankenhausstraße 12, 91054 Erlangen, Deutschland

**Keywords:** Lagerungsschäden, Qualitätsverbesserung, Fremdblut, Physiologische Kochsalzlösung, Hämofiltrationslösung, Cell Saving, Packed red blood cells, Storage lesion, Hemofiltration solution, Physiological saline solution

## Abstract

**Hintergrund:**

Die Transfusion von Erythrozytenkonzentraten (EK) ist mit verschiedenen Nebenwirkungen assoziiert, die u. a. durch Lagerungsschäden an Erythrozyten hervorgerufen werden. Die Zellen verändern ihre Struktur und setzen dabei Kalium sowie Lactat frei. Zur Minimierung dieser negativen Effekte können die Erythrozyten mithilfe einer maschinellen Autotransfusion (MAT) unter Verwendung von Waschlösungen aufgereinigt werden.

**Ziel der Arbeit:**

Untersuchung der Auswirkungen zweier Waschlösungen auf die gelagerten Erythrozyten.

**Material und Methode:**

In der vorliegenden Studie wurden 30 EK mittels MAT (Xtra, LivaNova, München, Deutschland) gewaschen. Der Goldstandard 0,9 %ige Kochsalzlösung (*n* = 15; N‑Gruppe) wurde mit einer 4 mmol/l kaliumhaltigen Hämofiltrationslösung (HF) (*n* = 15; HF-Gruppe; Duosol) verglichen. In einer Subgruppenanalyse wurde eine Differenzierung bezogen auf die Lagerdauer der EK (7, 14, 37 Tage) bis zur Durchführung der MAT vorgenommen. Untersucht wurde der Einfluss der Waschlösungen sowie des EK-Alters auf ATP, Lactat, Glucose, Elektrolyte und Zitrat zu drei Messzeitpunkten vor MAT (EKprä), unmittelbar danach (EKpost) und nach 10 h im Retransfusionsbeutel (EKpost10h).

**Ergebnisse und Diskussion:**

Die ATP-Konzentration nimmt durch die MAT-Waschung von EKprä zu EKpost signifikant zu (*n* = 30). Bei 37 Tage alten EK nimmt die ATP-Konzentration in der HF-Gruppe nach MAT stärker zu als in der N‑Gruppe. Durch die MAT-Waschung werden die Kalium‑, Lactat‑, Glucose- und Zitratkonzentration signifikant reduziert. Die MAT-Behandlung gelagerter EK verbessert deren Qualität. Das Waschen mit einer HF-Lösung führt zu einer physiologischeren Elektrolytzusammensetzung. Selbst 10 h nach MAT mit einer HF-Lösung ist die Qualität eines 37 Tage alten EK bezüglich der untersuchten Parameter mit einem jungen 7 Tage gelagerten, nichtgewaschenen EK vergleichbar.

**Zusatzmaterial online:**

Die Online-Version dieses Beitrags (10.1007/s00101-022-01189-6) enthält zusätzliches Material. Beitrag und Zusatzmaterial stehen Ihnen auf www.springermedizin.de zur Verfügung. Bitte geben Sie dort den Beitragstitel in die Suche ein, die Videos finden Sie beim Beitrag unter „Ergänzende Inhalte“.

## Abstract (en)

**Background:**

The transfusion of packed red blood cells (PRBC) is associated with various side effects, including storage damage to PRBCs. The cells change their structure, releasing potassium as well as lactate. Mechanical rinsing, available in many hospitals, is able to remove toxic substances and possibly minimizes the negative side effects of transfusion.

**Objective:**

The primary aim of our study was to improve the quality of PRBCs before transfusion. The effects of different washing solutions on PRBC quality were analyzed.

**Material and methods:**

This in vitro study compares 30 mechanically washed PRBCs. They were either processed with standard normal saline 0.9% (*n* = 15, N group) or a hemofiltration solution containing 4 mmol/l potassium (*n* = 15, HF group) by a mechanical rinsing device (Xtra, LivaNova, Munich, Germany). A subgroup analysis was performed based on the storage duration of the processed PRBCs (7, 14, 37 days). Samples were taken before washing (EKprä), immediately after washing (EKpost) and 10 h later (EKpost10h), after storage in the “wash medium” at room temperature. Concentrations of ATP (probability of survival in transfused erythrocytes), lactate, citrate and electrolytes (potassium, sodium, chloride, calcium) were tested.

**Results and conclusion:**

Mechanical rinsing improves pretransfusion quality of PRBC. Washing with a hemofiltration solution results in a more physiological electrolyte composition. Even 10 h after mechanical rinsing with a hemofiltration solution, the quality of 37-day-old PRBC is comparable to young PRBC that have been stored for 7 days and have not been washed. Washing stored PRBC increases the ATP content, which subsequently leads to an increased probability of survival of red cells after transfusion.

Die aktuelle Richtlinie Hämotherapie der Bundesärztekammer, aufgestellt im Einvernehmen mit dem Paul-Ehrlich-Institut, enthält neben allgemeinen Informationen zur Gewinnung von Blut und Blutbestandteilen und zur Anwendung von Blutprodukten einen speziellen Abschnitt 3.2.1.3 zu „gewaschenen Erythrozytenkonzentraten“ und die Abschnitte 2.6.4 und 3.2.5.3 zur maschinellen Autotransfusion. In einem geschlossenen System kann unter der Verwendung einer isotonischen Lösung das gelagerte Erythrozytenkonzentrat mehrfach gewaschen werden. Die anschließende Resuspendierung kann in einer isotonischen Kochsalzlösung oder alternativ in einer Additivlösung erfolgen [1].

## Hintergrund und Fragestellung

Das Waschen von autologem Blut in einem maschinellen Autotransfusionsgerät (MAT) wird im klinischen Alltag praktiziert. Die rechtlichen Auflagen zur Herstellung gewaschener Erythrozytenkonzentrate (EK) sind definiert, wenn das Verfahren mit einem geschlossenes MAT-Gerät durchgeführt wird. Der Schwerpunkt der vorliegenden Arbeit betrifft die Aufbereitung gelagerter homologer EK sowie die verwendeten Waschlösungen.

Im Allgemeinen ist die bestimmungsgemäße Durchführung der maschinellen Autotransfusion im klinischen Alltag sehr gut wissenschaftlich belegt. Entsprechend werden in Kliniken mit operativem Spektrum MAT-Geräte zur intraoperativen Saugerblutaufbereitung vorgehalten [2]. Das allgemeine Funktionsprinzip basiert auf der Zentrifugation, welche die einzelnen Bestandteile des Blutes nach ihrem Sedimentationskoeffizienten auftrennt. Die fertig aufbereiteten Erythrozyten stehen dann zur Retransfusion bereit. Als Vorgabe der MAT-Geräte-Hersteller wird als Waschlösung NaCl-Lösung, 0,9 %ig, aufgeführt. Diese Lösung besitzt eine unphysiologische Chlorid- und Natriumkonzentration von 154 mmol/l (Tab. 1). Eine übermäßige Infusion von 0,9 %iger NaCl-Lösung kann die Plasma-Ionen-Konzentration von Na und Cl erhöhen, sodass eine metabolische Acidose die Folge sein kann [3]. Durch die Infusion größerer Mengen dieser „physiologischen Kochsalzlösung“ kann es zu einer renalen Vasokonstriktion kommen, einer verminderten Miktion und einer erhöhten Inzidenz einer Niereninsuffizienz [4]. Verschiedene Hersteller von MAT-Geräten haben die „physiologische 0,9 %ige Kochsalzlösung“ trotz möglicher Nebeneffekte in ihren Bedienungsanleitungen als alternativlosen „Goldstandard“ definiert [5]. Andere Hersteller, wie z. B. LivaNova, erlauben zusätzlich auch andere Lösungen, welche „für intravenöse Anwendungen zugelassen sind“ [6].

**Table 1:** 

Elektrolyt/Metabolit	Einheit	0,9 %ige NaCl-Lösung	Hämofiltrationslösung (Duosol; B. Braun)	Blutplasma	Vollelektrolytlösung (Jonosteril)
Na	mmol/l	154	140	136–146	137
K	mmol/l	0	4	3,5–5,0	4
Cl	mmol/l	154	113	98–106	110
Ca	mmol/l	0	1,5	1,15–1,29	1,65
Mg	mmol/l	0	0,5	0,7–1,6	1,25
Osmolarität	mosmol/l	308	300	280–300 (mosmol/kg)	291
pH	–	5–7	7–8	7,35–7,45	5–7
HCO_3_^−^	mmol/l	0	35	22–26	0
CH_3_OO^−^	mmol/l	0	0	0	36,8
Glucose	mg/dl	0	100	70–105	0

Acetat anion (CH3COO^−^)

Osmotische Konzentration (mosmol/kg)

### Nachbehandlung gelagerter Erythrozyten

Es gibt in der Literatur mehrere Ansätze zur Nachbehandlung von gelagerten EK, um die negativen Effekte der nichtvermeidbaren Lagerungsschäden zu reduzieren. Neben verschiedenen neuartigen Additivlösungen steht die MAT-Behandlung vor einer Transfusion zur Verfügung. Für die EK-Vorbehandlung wurden von verschiedenen Autoren alternative Waschlösungen getestet. Yang et al. versuchten eine Mannitol-Adenin-Phosphat-Lösung zur Waschung und zur Stabilisierung [7]. Die von ihnen verwendete Stabilisatorlösung konnte das bearbeitete EK u. a. länger haltbar machen. Huber et al. wählten erstmals den Ansatz einer „bikarbonatgepufferten“ Hämofiltrationslösung ohne Kalium zur MAT-Behandlung von EK mit ausschließlich positivem Effekt [8]. Gezeigt werden konnte, dass es neben der Verbesserung des Säure-Base-Haushaltes im Lagermedium zu einer verbesserten Stabilität der Erythrozyten kam.

In Bezug auf die erforderliche Elektrolytzusammensetzung für eine Volumentherapie sind aktuell zwei Richtlinien von klinischer Bedeutung. Zum einen die aktuelle S3-Leitlinie zur intravasalen Volumentherapie bei Erwachsenen, zum anderen die S1-Leitlinie perioperative Infusionstherapie bei Kindern. Beide Leitlinien empfehlen grundsätzlich die Verwendung von balancierten Vollelektrolytlösungen zur Volumentherapie [9, 10].

### Gesetzliche Rahmenbedingungen der MAT

Den gesetzlichen Rahmen für die Transfusion von Erythrozytenkonzentraten bildet das Transfusionsgesetz [11]. Hierin wird neben allgemeinen Regelungen des Transfusionswesens auf die Details in Richtlinien nach §§ 12a und 18 TFG verwiesen. In der entsprechenden Hämotherapie-Richtlinie von 2017 haben die Bundesärztekammer und die zuständige Bundesoberbehörde, das Paul-Ehrlich-Institut (PEI), im Abschnitt 3.2.1.3 die Anforderungen an das Waschen gelagerter EK näher definiert [1]. Das Aufbereiten eines gelagerten EK hat in einem geschlossenen System zu erfolgen. Als Lagermedium nach dem Aufbereiten mit 0,9 %iger NaCl-Lösung sind auch Additivlösungen erlaubt. Gewaschen werden dürfen nur EK, die den allgemeinen Anforderungen der Richtlinie entsprechen. Die anschließende Lagerungsdauer hängt von der durchgeführten Validierung des Prozesses ab, in dem der Hämatokrit, das Gesamt-Hb, die Hämolyserate und der Proteingehalt bestimmt werden. Darüber hinaus muss eine mikrobiologische Kontrolle durchgeführt werden.

### Gelagerte Erythrozyten

Aus dem Metabolismus der Erythrozyten erklärt sich ihre Schädigung durch die Lagerung. In der Zellreifung präzipitieren die Mitochondrien. Durch den funktionellen Verlust der Mitochondrien stehen der kernlosen Zelle β‑Oxidation, der Zitratzyklus und die Atmungskette zur Energiegewinnung nicht mehr zur Verfügung. Die anaerobe Glykolyse wird zur Energiegewinnung herangezogen. Diese führt zum Endprodukt Lactat, das in vivo außerhalb der Erythrozyten weiterverstoffwechselt werden kann, sich im Blutbeutel aber kumuliert und zur zunehmenden Ansäuerung im EK führt. Hierdurch kommt es zum Nachlassen der Glykolyse; dies führt zu einer verringerten Verfügbarkeit von ATP. Aus dem ATP-Mangel resultiert, dass der aktive Ionentransport nicht mehr vollumfänglich gewährleistet werden kann. Hieraus ergeben sich intrazellulär ein verminderter Kaliumgehalt sowie eine erhöhte Natrium- und Kalziumkonzentration. Darüber hinaus nimmt durch den ATP-Mangel die Synthese bzw. die Reduktion von Glutathion ab. Das Tripeptid ist Hauptbestandteil des Glutathionredoxsystems. In seiner reduzierten Form schützt es die Sulfhydrylgruppen von Enzymen, Proteine der Erythrozytenmembran und das Hämoglobin vor der Oxidation. Insgesamt bleibt allerdings die antioxidative Kapazität während der ganzen Lagerungsdauer ausreichend [12]. Neben diesen genannten Effekten kann es im Laufe der Alterungs- und Lagerungszeit auch zu einer Zunahme von intrazellulärem Wasser kommen, wodurch die Hämoglobinkonzentration in der Zelle sinkt und die wichtige Deformierbarkeit abnimmt. Zusätzlich wechselt Phosphatidylserin die Position von der Innenseite zur Außenseite und ist somit Angriffspunkt für Makrophagen. Wegen der genannten Zusammenhänge wird die ATP-Konzentration für die qualitative Beurteilung von EK herangezogen. Heiden et al. definieren die ATP-Konzentration sogar als den wichtigsten Surrogatmarker für die Überlebenswahrscheinlichkeit gelagerter Erythrozyten im Transfusionsempfänger [13].

Die vorliegende Arbeit beschäftigt sich mit der Fragestellung der Qualitätsverbesserung gelagerter Erythrozytenkonzentrate (EK) mittels eines maschinellen Autotransfusionsgerätes. Untersucht wurde, ob die Wahl der verwendeten Waschlösung bei der maschinellen Autotransfusion einen Einfluss auf die Qualität der Erythrozyten hat und das aufbereitete EK dadurch verbessert wird.

## Studiendesign und Untersuchungsmethoden

Die In-vitro-Studie wurde von der Ethik-Kommission der FAU mit dem Ethikvotum Nr.: 22-41-ANF als unbedenklich bewertet. Die verwendeten EK wurden von der Transfusionsmedizinischen und Hämostaseologischen Abteilung des Universitätsklinikums Erlangen, welche die gesetzlichen und klinikinternen Vorgaben zu Herstellung, Prüfung und Lagerung von EK erfüllt, zur Verfügung gestellt. Dreißig leukozytendepletierte „inline“-gefilterte EK in einer Phosphat-Adenin-Glucose-Guanosin-Saline-Mannitol-Stabilisierungslösung (PAGGS-M) wurden in vitro untersucht. Die Studie wurde unter der arzneimittelrechtlichen Verantwortung des beteiligten Transfusionsmediziners durchgeführt.

### Versuchsaufbau und Versuchsablauf

#### Detaillierter Studienablauf im digitalen Supplement

**Figure 1:**
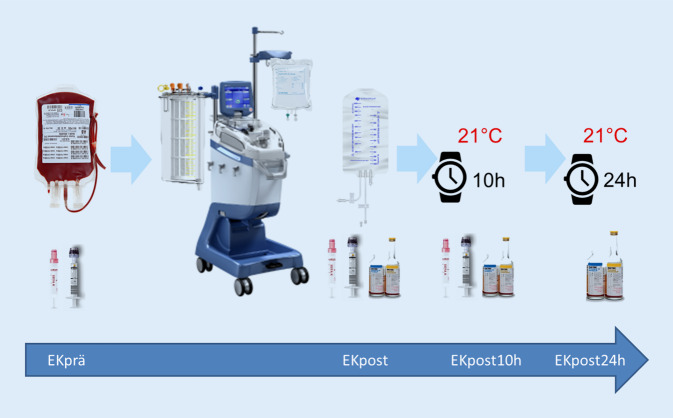

Für die In-vitro-Studie wurde ein MAT-Gerät (Xtra; LivaNova) mit dem standardisierten Waschprogramm „Popt“ verwendet. Für die Untersuchung der unterschiedlichen MAT-Waschlösungen wurden die 30 EK in zwei Gruppen geteilt: Gruppe N (*n* = 15) = NaCl-Lösung, 0,9 %ig, 3000 ml (Fresenius, Fresenius Kabi AG, Deutschland) vs. Gruppe HF (*n* = 15) = Hämofiltrationslösung (Duosol 5000 ml, B. Braun, Deutschland). In einer Subgruppenanalyse erfolgte eine weitere Differenzierung bezogen auf die Lagerungsdauer der EK (7, 14, 37 Tage je 5 pro Gruppe). Gewaschen wurden alle EK nach Studienprotokoll mit 1000 ml Waschlösung. Proben wurden zu vier Messzeitpunkten entnommen: EKprä (vor MAT-Waschung), EKpost (nach MAT-Waschung), EKpost10h (10 h Lagerung im Retransfusionsbeutel bei Raumtemperatur) und EKpost24h (24 h Lagerung im Retransfusionsbeutel bei Raumtemperatur) (Abb. 1).

Zur Bestimmung des ATP-Gehalts wurden die Blutproben zentrifugiert und der proteinfreie Überstand abpipettiert, in CryoTubes (Thermo Fisher Scientific) überführt und anschließend bei −80 °C für die nachfolgenden En-bloc-Messungen gelagert. Diese quantitative In-vitro-Bestimmung von ATP wird mit der Hexokinasemethode an fotometrischen Systemen (Kit-Nr. 1 6201, DiaSys GmbH) durchgeführt. Die Plasmaproben wurden mittels einem automatisierten Analysegerät (Cobas 6000, Roche) ausgewertet; neben den Elektrolyten (Na, K, Cl und Ca) erfolgte die Konzentrationsbestimmung der beiden Metabolite Lactat und Glucose. Im gleichen Zug wurden für den Säure-Basen-Haushalt das pCO_2_, das HCO_3_^−^, der BE und pH bestimmt.

### Mikrobiologische Untersuchung

Im Rahmen der Studie wurden mikrobiologische Untersuchungen durchgeführt. Hierzu wurden zu den Messzeitpunkten EKprä, EKpost und EKpost24h aus dem bei Raumluft gelagerten Retransfusionsbeutel je 16 ml aufbereitetes EK entnommen. Zur Beimpfung der aeroben und anaeroben Blutkulturflaschen wurden hierbei jeweils 8 ml EK/Kulturflasche benötigt.

### Statistik

Die statistische Auswertung erfolgte mit SPSS für Windows (Version 21 SPSS Inc., USA). Der Test auf Normalverteilung erfolgte mittels Q‑Q-Plot. Die gemessenen Werte zu einem bestimmten Messzeitpunkt wurden mittels Welch *t*-Test ausgewertet. Die Analyse zwischen den einzelnen Messzeitpunkten erfolgte mittels gepaartem *t*-Test. Alle ermittelten Werte sind in der Form Mittelwert ± einfache Standardabweichung angegeben. Das Signifikanzniveau betrug α = 0,05.

## Ergebnisse

### ATP

**Figure 2:**
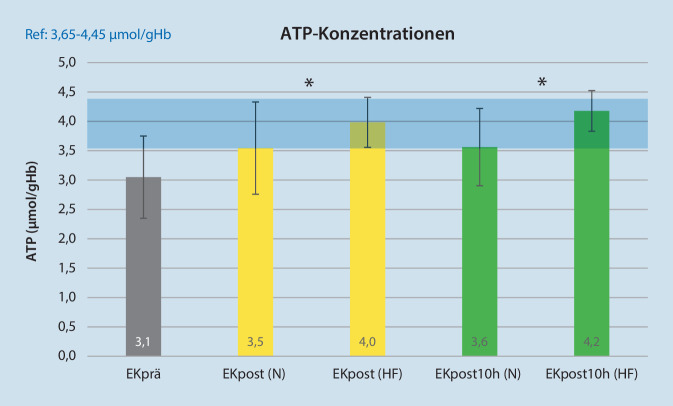

Der ATP-Gehalt aller EK nimmt von Messpunkt EKprä zu EKpost signifikant zu (*n* = 30; EKprä: 3,1 ± 0,7 µmol/gHb vs. EKpost: 3,8 ± 0,7 µmol/gHb; *p* < 0,05). Die Abb. 2 zeigt die ATP-Konzentration nach dem Prozessieren und der 10-stündigen Lagerung, unterteilt nach der jeweils verwendeten Waschlösung. In der Gruppe der 37 Tage alten EK nimmt die ATP-Konzentration in der HF-Gruppe im Verhältnis zur N‑Gruppe signifikant zu (EKpost vs. EKpost10h: 3,7 ± 0,4 vs. 4,0 ± 0,5 µmol/gHb; *p* < 0,05).

### Elektrolyte und Metaboliten

**Figure 3:**
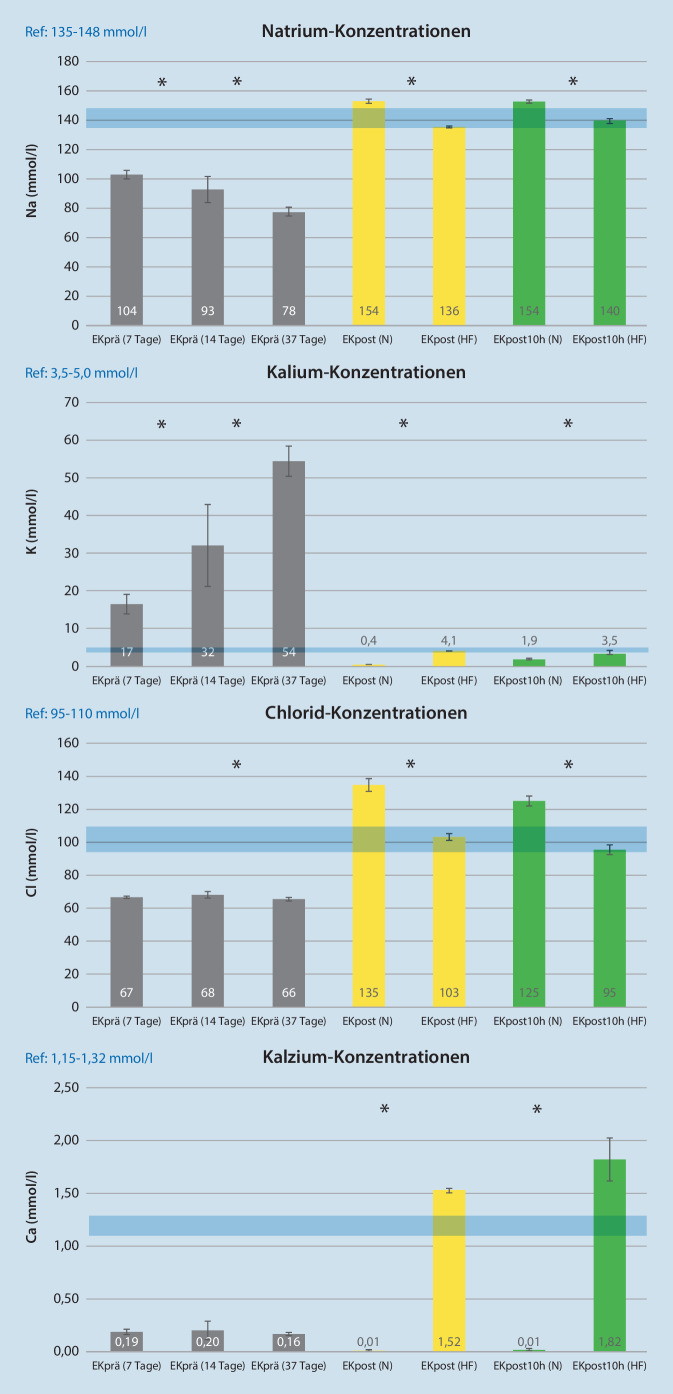

Zum Zeitpunkt EKprä lagen alle gemessenen Elektrolyte (Na, K, Cl und Ca) unabhängig von der Lagerungsdauer in unphysiologischen Konzentrationsbereichen (EKprä) (Abb. 3). In den EK der HF-Gruppe liegen alle Elektrolyte nach dem Waschen (EKpost) in einem physiologischeren Bereich. Der Messzeitpunkt EKpost zeigt in der N‑Gruppe im Vergleich zur HF-Gruppe einen signifikanten Unterschied bezogen auf die Natrium- (154 mmol/l vs. 136 mmol/l) und Chloridkonzentration (135 mmol/l vs. 103 mmol/l). In der HF-Gruppe nimmt die Natriumkonzentration zwischen den Zeitpunkten EKpost und EKpost10h signifikant auf physiologische Werte zu (Na = 140 mmol/l), während sie in der N‑Gruppe auf einem unverändert unphysiologischen hohen Niveau bleibt. Die Ausgangskaliumkonzentration, die über die Lagerdauer (EKprä) zunimmt, sinkt durch die MAT (EKpost) in beiden Gruppen signifikant. Im physiologischen Referenzbereich liegt die HF-Gruppe zum Zeitpunkt EKpost mit 4,1 mmol/l, während die N‑Gruppe diesen mit 0,4 mmol/l unterschritt. In der HF-Gruppe führt die offensichtlich funktionierende Natrium-Kalium-Pumpe in den Erythrozyten bis zum Zeitpunkt EKpost10h zu einer signifikanten Abnahme der Kaliumkonzentration (3,5 mmol/l). Die Kalziumkonzentration in den gelagerten EK (EKprä) war unabhängig von der Lagerungszeit mit < 0,2 mmol/l sehr niedrig. Zum Messzeitpunkt EKpost und EKpost10h konnte kein Kalzium mehr in der N‑Gruppe nachgewiesen werden. In der HF-Gruppe kam es zu einem signifikanten Anstieg nach dem Waschen und einem weiteren Anstieg bis zu EKpost10h (Abb. 3).

**Figure 4:**
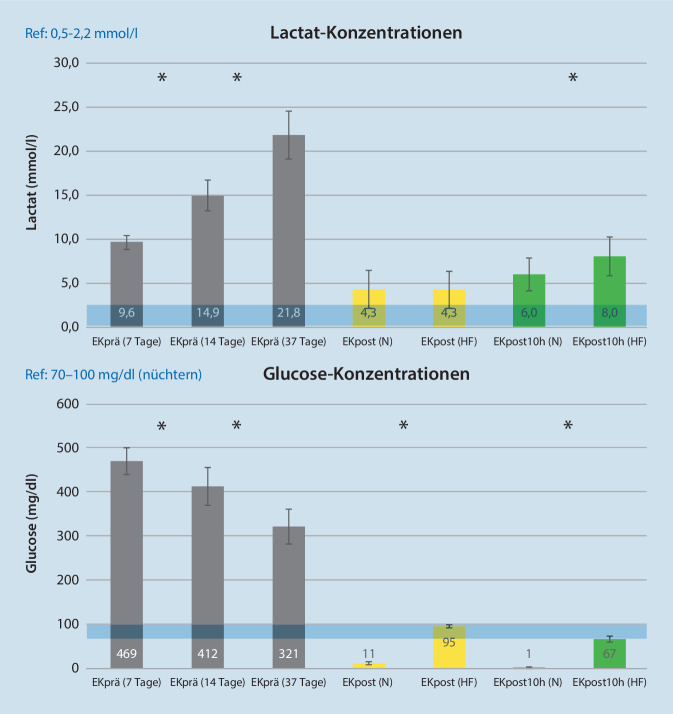

Die unphysiologische Glucosekonzentration in gelagerten EK vor MAT-Waschung ist von der Lagerungsdauer abhängig und konnte in der N‑Gruppe fast vollständig durch die MAT-Behandlung ausgewaschen werden (11 mg/dl). In der HF-Gruppe kam es zum Zeitpunkt EKpost zu einer Reduktion der Glucose auf ein physiologisches Niveau (95 mg/dl). Durch den noch funktionierenden Metabolismus der Erythrozyten kam es zu einer weiteren signifikanten Reduktion in der HF-Gruppe auf 67 mg/dl vs. 1 mg/dl in der N‑Gruppe (Abb. 4). Die Lactatkonzentration nimmt über die Lagerungszeit signifikant zu und nimmt durch die MAT wieder signifikant ab. Nach 10 h Lagerung liegt in der HF-Gruppe eine signifikant höhere Konzentration vor (Abb. 4).

### Säure-Base-Haushalt

**Figure 5:**
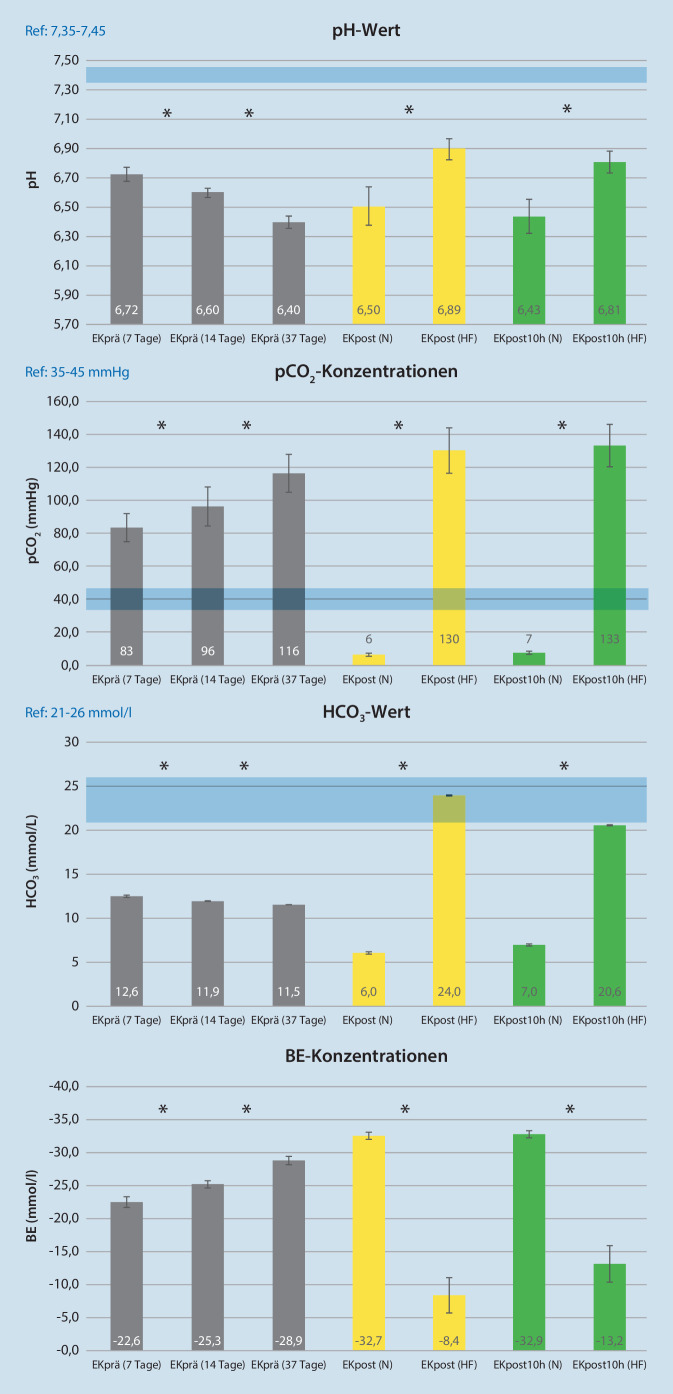

pH-Wert, HCO_3_^−^ und BE nehmen über die Lagerungszeit (7 bis 37 Tage) signifikant (*p* < 0,05) ab, bei signifikant höherem pCO_2_ (*p* < 0,05). Betrachtet man den pH-Wert nach der MAT-Aufbereitung bezogen auf die Waschlösung, stellt man fest, dass in der HF-Gruppe insgesamt ein höherer pH-Wert unabhängig von der Lagerzeit auftritt (N-Gruppe vs. HF-Gruppe: pH-Wert EKpost: 6,50 vs. 6,89) und nur leicht über die post-MAT Lagerzeit abnimmt (EKpost10h: 6,43 vs. 6,81) (Abb. 5). Bezogen auf den pCO_2_ und BE wurde sowohl zum Zeitpunkt EKpost als auch zum Zeitpunkt EKpost10h in der HF-Gruppe signifikant erhöhte pCO_2_-Werte bei sinkenden BE und in der N‑Gruppe ein gegensätzliches Verhalten festgestellt. Im Mittel, über alle EK (*n* = 30), sehen wir einen HCO_3_^−^-Wert von 12 mmol/l. Waschlösungsbedingt verändert sich der HCO_3_^−^ nach der Aufbereitung (EKpost) signifikant unterschiedlich (N-Gruppe vs. HF-Gruppe: HCO_3_^−^-Wert: 6 mmol/l vs. 24 mmol/l) (Abb. 5).

### Zitrat

Die mittlere Zitratkonzentration im Lagermedium bei 30 EK betrug 2,72 ± 0,44 mmol/l zum Messzeitpunkt EKprä. Die Konzentration zum Messzeitpunkt EKpost nach MAT-Behandlung betrug unabhängig von der Waschlösung 0,0 mmol/l.

### Mikrobiologische Untersuchung

In den insgesamt 180 Blutkulturen wurden zu bei acht verschiedenen Proben unterschiedlichen Zeitpunkten Hautkeime (*Cutibacterium acnes*; *Staphylococcus haemolyticus*; *Microbacterium aurum*; *Corynebacterium simulans*) gefunden.

## Diskussion

Der Adenosintriphosphatgehalt in den Erythrozyten ist von elementarer Bedeutung, da er mit der Überlebenswahrscheinlichkeit der Erythrozyten nach der Transfusion korreliert [13]. Durch eine übermäßige Abnahme des ATP-Gehalts kommt es zu einer morphologischen Verformung der Zellen hin zu Echinozyten. Diese strukturellen Veränderungen machen die deformierten Erythrozyten im Transfusionsempfänger zum Ziel von Makrophagen und fördern damit deren rasche Elimination aus dem Kreislauf. Stewart et al. konnten zeigen, dass die CD47-Expression auf den Erythrozyten mit der Lagerungsdauer abnimmt [14]. Zusätzlich führt eine Abnahme der ATP-Konzentration zu einer verminderten Deformierbarkeit der Zellen und damit zu einer schlechteren Mikrozirkulation nach der Transfusion [15]. Daher empfiehlt die Zulassungsbehörde, den ATP-Gehalt als Prüfkriterium für die Qualität von gelagerten EK heranzuziehen [13]. Der signifikante Zunahme der ATP-Konzentration in den EK nach dem Waschen in der vorliegenden Studie lässt sich durch die MAT erklären. Das lactatvermittelte saure Milieu um die Erythrozyten wird beseitigt, und die Enzyme der Erythrozyten können wieder in einem physiologischeren Milieu arbeiten. Die retransfundierten aufbereiteten Erythrozyten haben durch den höheren ATP-Gehalt nach der Transfusion im Patienten eine höhere Überlebenswahrscheinlichkeit. Vergleicht man beide Studiengruppen, fällt die signifikant unterschiedliche Glucosekonzentration nach MAT auf. Damit fehlt in der N‑Gruppe den gewaschenen Erythrozyten das grundlegende Substrat für die anaerobe Glykolyse, die in der HF-Gruppe in ausreichender Menge für die Verstoffwechslung zur Verfügung steht. Somit nehmen in der HF-Gruppe der ATP-Gehalt und damit die Vitalität der Erythrozyten während der 10 h Lagerung sogar wieder zu.

Über die Lagerungszeit von EK nimmt die Glucosekonzentration, wie dargestellt, ab (Abb. 4), gleichzeitig steigt Lactat als Produkt der anaeroben Glykolyse an (Abb. 4). Dieses Phänomen und der direkte Zusammenhang mit der anaeroben Glykolyse konnten nachgewiesen werden [16]. Durch eine MAT wird das kumulierte Lactat signifikant ausgewaschen. Im Verlauf der nachfolgenden 10-stündigen Lagerung bei Raumtemperatur zeigte sich ein wiedereinsetzender Metabolismus, welcher einen Wiederanstieg der Lactatkonzentration zur Folge hat. In der HF-Gruppe kumulierte sich hierdurch das anfallende Lactat stärker als in der N‑Gruppe. Es sei hier nochmals darauf hingewiesen, dass in der N‑Gruppe der Metabolismus durch die fast komplett fehlende Glucose eine anaerobe Glykolyse faktisch nur eingeschränkt möglich ist. Dieses Hintergrundwissen ist essenziell, um die dargestellten absoluten Lactatkonzentrationen nach der Wiederaufbereitung richtig einzuordnen. Betrachtet man den maximalen Lactatwert eines 37 Tage gelagerten und dann mit MAT behandelten EK zum Zeitpunkt EKpost10h (HF-Gruppe), so ist das gemessene Lactat mit einem 7 Tage alten ungewaschenen EK vergleichbar, sodass man von einer „Verjüngung“ um 30 Tage ausgehen könnte. Die mit zunehmender Lagerung der Erythrozyten nachlassende Aktivität wichtiger pH-abhängiger Enzyme kann durch die MAT wiederhergestellt werden.

Der pH-Wert nimmt im EK über die Lagerungsdauer kontinuierlich ab. Sümpelmann et al. konnten zeigen, dass es zwischen Lagerdauer (1 bis 17 Tage) und pH-Wert (6,53–6,99) eine starke Korrelation besteht [17]. Die Grenze zum vollständigen Funktionsverlust der an der Glykolyse beteiligten Enzyme liegt bei einem pH von 6,4. Diese ist bei 37 Tage gelagerten EK mit 6,4 noch eingehalten. Durch MAT mit gepufferter HF-Lösung kann der pH-Wert in einem 37 Tage alten EK auf 6,8 angehoben werden. In der N‑Gruppe fällt der pH nach MAT auf 6,35 ab. Dies könnte, neben dem fast vollständigen Fehlen der Glucose, eine weitere Ursache für das erniedrigte ATP nach Lagerung in 0,9 %iger NaCl-Lösung sein. Der Unterschied des pH-Werts ist gruppenspezifisch auf die verwendete Waschlösung zurückzuführen. Bei der Bikarbonat (HCO_3_^−^) gepufferten Waschlösung steigt das pCO_2_, wohingegen die 0,9 %ige NaCl-Waschlösung dem aufzubereitenden EK HCO_3_^−^ entzieht. Zander et al. konnten diese HCO_3_^−^-Absenkung bei unphysiologischer Waschlösung ebenfalls nachweisen [18].

Den Einfluss des HCO_3_^−^ konnte auch in der tierexperimentellen Studie von Sümpelmann et al. nachgewiesen werden. Hierbei wurde der Einfluss der gewählten Waschlösung auf den Säure-Base-Haushalt der Tiere bei einer Massentransfusion untersucht. Es zeigte sich, dass durch 0,9 %ige NaCl-Lösung als Waschlösung eine Dilutionsacidose entsteht. Dieses Phänomen konnte in der HCO_3_^−^-gepufferten Waschlösung nicht nachgewiesen werden [19].

Nach einem Abfall des pH-Werts in den Erythrozyten wird die Natrium-Kalium-Pumpe inaktiviert, und bedingt durch die Lagerzeit kumulieren Stoffwechselendprodukte im EK [20, 21]. Die Hämolyse eines Teils der Erythrozyten steigert noch die durch Funktionsverlust der Natrium-Kalium-Pumpe bedingte extrazelluläre Kumulation von Kalium im gelagerten EK (Abb. 3).

Durch eine Hämolyse kommt es zum Anstieg der Konzentrationen vom freien Eisen und freiem Hb. Im Körper zirkulierendes freies Hb kann zu einer erhöhten Gefäßpermeabilität mit Endotheldysfunktion führen [22]. Freies Hämoglobin interagiert um einen Faktor 1000 schneller mit Stickstoffmonoxid (NO) im Vergleich zu Erythrozyten, was zu einer Vasokonstriktion mit verschlechterter Mikrozirkulation führen kann [23]. Zusätzlich wird das freie Hb als möglicher Mediator bei der Entstehung eines „acute respiratory distress syndrome“ (ARDS) angesehen [24]. Refaai et al. konnten zeigen, dass sich mittels MAT auch das freie Hb signifikant absenken lässt [25]. Vorgeschädigte ältere Erythrozyten von minderer Qualität sowie bereits tote Zellen werden im Zuge einer positiven Selektion ebenfalls ausgewaschen.

Für die Mehrzahl der Patienten, denen EK transfundiert werden, dürfte die tatsächliche klinische Bedeutung der gravierenden Veränderungen, die Erythrozyten während der Lagerung erleiden, gering sein [26, 27].

Erhöhtes extrazelluläres Kalium kann aber bei pädiatrischen herzchirurgischen Patienten beim Starten einer mit EK vorgefüllten (geprimten) Herz-Lungen-Maschine zu einer akuten transfusionsassoziierten Hyperkaliämie mit daraus resultierendem Herzstillstand führen [28]. Swindell et al. untersuchten diesen Effekt an Neugeborenen, die zur Hälfte gewaschene EK erhielten, und in einer Kontrollgruppe mit ungewaschenen EK. Eine transfusionsassoziierte Hyperkaliämie trat bei 4 der 11 Patienten nach der Transfusion von ungewaschenen EK auf, was bei 2 Patienten zu einem Kammerflimmern führte [29]. Osthaus et al. zeigten, dass bei einem pädiatrischen Herz-Lungen-Maschinen-Einsatz die Verwendung einer physiologischen Restitutionslösung in Form einer HCO_3_^−^-gepufferten Hämofiltrationslösung Störungen des Elektrolyt- und Säure-Base-Haushalts verringern kann [30]. In den „Guidelines for Pediatric and Congenital Perfusion Practice“ der American Society of Extracorporeal Technology wird empfohlen, bei Verwendung von EK zum Priming entweder eine Ultrafiltration durchzuführen oder gewaschene EK zu verwenden [31].

### Elektrolytveränderungen

Durch das Waschen eines EK erfolgt eine Veränderung der Elektrolytzusammensetzung vom „ursprünglichen“ zum „neuen“ Lagermedium. Dabei ist die Art der verwendeten Waschlösung bestimmend für die Elektrolytzusammensetzung im Retransfusionsbeutel. Die Verwendung einer Hämofiltrationslösung führt aufgrund ihrer Zusammensetzung zur Abnahme von elektrolytassoziierten Nebenwirkungen. Vergleicht man die in der vorliegenden Arbeit verwendeten Waschlösungen in Bezug auf Natriumionen, fällt die unphysiologisch hohe Na-Konzentration der Waschlösung in der N‑Gruppe mit 154 mmol/l vs. 140 mmol/l in der HF-Gruppe auf. Nach dem Waschen des Blutes zeigt sich, dass alle gemessenen Elektrolyte nur in der HF-Gruppe in einem physiologischen Bereich liegen. Die negativen Einflüsse nach Massivinfusion mit (un)physiologischer Kochsalzlösung kann die Plasmaionenkonzentration von Na^+^ und Cl^−^ erhöhen, sodass eine metabolische Acidose die Folge ist [3]. Diese kann zu einer renalen Vasokonstriktion mit verminderter Diurese und einer akuten Niereninsuffizienz (ANI) führen [32]. Ursächlich für eine ANI könnte hierbei ein erhöhte Chloridkonzentration der transfundierten Kochsalzlösung sein. Proffitt et al. konnten bei 14 Tage alten EK, die mit 0,9 %iger NaCl-Lösung gewaschen wurden, eine signifikant höhere Hämolyserate im Vergleich zu EK, die mit SAGM gewaschen wurden, feststellen [33].

### Mikrobiologische Untersuchung

In den Ergebnissen der 180 Blutkulturen wurden 8 positive Keimnachweise gefunden. Verunreinigungen durch die Behandlung der EK mit MAT konnten nicht nachgewiesen werden. Zur Beimpfung der aeroben und anaeroben Blutkulturflaschen wurden die Kappe der Kulturflasche ohne Handschuhe geöffnet und mit Blut aus dem Retransfusionsbeutel befüllt. Hierbei dürften Hautkeime vom Laborpersonal übertragen worden sein. Das gefundene Keimspektrum zeigte ausnahmslos Hautkeime. Es gab kein einziges EK, in dem sich bei weiterführenden Messzeitpunkt des gleichen Retransfusionsbeutel positive Keimnachweise fanden. Da kein weiteres Keimwachstum nachweisbar war, ist in jedem Fall einer positiven mikrobiologischen Kontrolle von unsachgemäßer Handhabung beim Beimpfen der Kulturflaschen auszugehen.

### Arzneimittelrechtliche Aspekte

Arzneimittelrechtlich wirft das Waschen von homologen EK mittels einer MAT-Maschine unmittelbar vor dem Priming einer Herz-Lungen-Maschine mit den so gewonnenen gewaschenen EK in Deutschland Probleme auf. Auch bei Verwendung zugelassener und vom Hersteller in Verkehr gebrachter EK stellt jeder nachgelagerte Bearbeitungsschritt, sei es Teilung, Bestrahlung oder eben Waschen, einen Schritt dar, der den weit gefassten Begriff Arzneimittelherstellung im Sinne von § 4 Abs. 14 AMG anwendbar macht. Da es sich im beschriebenen Kontext um eine patientenbezogene gerichtete Herstellung handelt und kein Inverkehrbringen seitens der Person, die die MAT durchführt, vorliegt, wenn sie selbst die entstehenden gewaschenen EK zum Priming einsetzt, ist eine Herstellungserlaubnis nach § 13 AMG nicht erforderlich. Sehr wohl unterliegt die Tätigkeit aber der allgemeinen Anzeigepflicht nach § 67 AMG. Offen ist aus unserer Sicht dagegen, ob die beschriebene Tätigkeit des Waschens homologer EK per se eine Spendeeinrichtung im Sinne von § 2 Nr. 1 TFG eröffnet. Diese Frage stellt sich natürlich nicht, wenn die Einrichtung auch die klassische MAT als fremdblutsparendes autologes Hämotherapieverfahren einsetzt, wovon in der Regel auszugehen sein dürfte. Gerade dann ist aber die Frage, ob der Qualifikationsnachweis des Facharztes für Anästhesiologie als leitende ärztliche Person i. S. v. § 4 S. 1 Nr. 2 TFG, den Abschnitt 2.6.4 der Richtlinie Hämotherapie erlaubt, auch diese Sonderanwendung der MAT abdecken kann [1]. Da unseres Erachtens die aktuellen Fassungen von AMG, TFG und Richtlinie Hämotherapie keine zweifelsfreie Antwort geben, dürfte die zuständige Aufsichtsbehörde einen Ermessensspielraum haben.

Hierbei sollte berücksichtigt werden, dass derzeit keine gute Alternative zur Vermeidung lebensgefährlicher Arrhythmien in der Kinderherzchirurgie durch anflutendes Kalium aus zum Priming der Herz-Lungen-Maschine eingesetzten EK vorhanden ist. Die systematische Bestellung gewaschener EK von versorgenden Spendediensten als Alternative beinhaltet erhebliche Probleme hinsichtlich Logistik und zeitnaher Bereitstellung benötigter Präparate.

### Weitere positive Nebeneffekte durch MAT anhand ausgewählter Studien

Erythrozyten Konzentrate werden in der Regel in weichmacherhaltigen PVC-Lagerbeuteln bei 4 °C aufbewahrt. Mehrere Studien konnten nachweisen, dass durch das Waschen von EK mittels MAT unabhängig von der Waschlösung der primäre Weichmacher (DEHP) sowie sein direkter Metabolit (MEHP) signifikant reduziert werden konnten [34, 35]. Bezogen auf die Elimination von Heparin aus der MAT-Spüllösung konnten Kwapil et al. feststellen, dass beim Waschen von EK eine um 99,6 %ige Reduktion des initial zugesetzten Heparins (25000 IE) erzielt werden konnte. Die Arbeitsgruppe konnte somit nachweisen, dass die Restheparinmenge pro transfundiertem Volumen unterhalb einer therapeutischen Wirksamkeit liegt [36]. Durch die MAT kommt es zur vollständigen Elimination von Zitrat, welches dem „ursprünglichen“ Lagermedium zugesetzt wurde. Die Zitratelimination bietet den Vorteil, dass keine Metabolisierung in der Leber stattfindet und das Risiko einer Posttransfusionsalkalose minimiert ist [18].

### Limitationen

Die Verwendung von 150 mm Hg Vakuum muss als Limitation der Studie betrachtet werden. Durch diesen Unterdruck kann es zu Hämolyse durch die hohen Scherkräfte beim Einsaugen ins Reservoir kommen. Die Schwelle des negativen Drucks beträgt laut Jegger et al. 120 mm Hg [37]. Bezogen auf das gewaschene Endprodukt im Retransfusionsbeutel, solle diese Limitation ohne Bedeutung sein, da das freie Hb durch das Waschen signifikant gesenkt wird [25].

Beim Beimpfen der Kulturflaschen zur mikrobiologischen Beurteilung der MAT-Waschung kam es in 4,4 % der Fälle zu laborpersonalbedingten Verunreinigungen.

## Schlussfolgerung

Durch die Verwendung einer glucosehaltigen Hämofiltrationslösung mit 4 mmol/l Kalium als Waschlösung ist es möglich, die Qualität von gelagerten EK nach MAT signifikant zu verbessern. Durch das Waschen gelagerter EK nimmt der ATP-Gehalt in den aufbereiteten Erythrozyten zu, was in der Folge zu einer erhöhten Überlebenswahrscheinlichkeit bei retransfundierten Erythrozyten führt. Der erhöhte pH-Wert in der MAT-Gruppe mit einer glucosehaltigen Hämofiltrationslösung führt zu vermehrten Zellaktivitäten mit Aktivierung der Natrium-Kalium-Pumpe. Die lagerungsbedingten Elektrolytverschiebungen werden durch die Hämofiltrationslösung „neutralisiert“, nach dem Waschen sowie nach 10 h Lagerung im Retransfusionsbeutel befinden sich die Elektrolytwerte in einem physiologischen Bereich.

## Fazit für die Praxis

- Eine isotonische Hämofiltrationslösung mit Glucose als Waschlösung gewährleistet, dass den Erythrozyten nach der Aufbereitung mittels geschlossenem MAT-Gerät genügend Glucose bei höherem pH für die anaerobe Glykolyse zur Verfügung steht.
- Abhängig von der Waschlösung ist das Elektrolytspektrum im gewaschenen EK physiologisch.

## Supplementary Information




