# Supplementary material for: Improved quality of stored packed red blood cells by mechanical rinsing
Source: Anaesthesiologie. 2022 Aug 15;71(11):882–92. [Article in German] doi: 10.1007/s00101-022-01189-6 (PMC9636120; doi:10.1007/s00101-022-01189-6)
Supplement: Supplementary file 1 [file 101_2022_1189_MOESM1_ESM.pdf]

Detaillierter Studienablauf im digitalen Supplement zum Artikel mit dem Titel:

Verbesserte Qualität gelagerter Erythrozyten Konzentrate durch Maschinelle Autotransfusion

Münch et al.

Untersuchungsmethoden

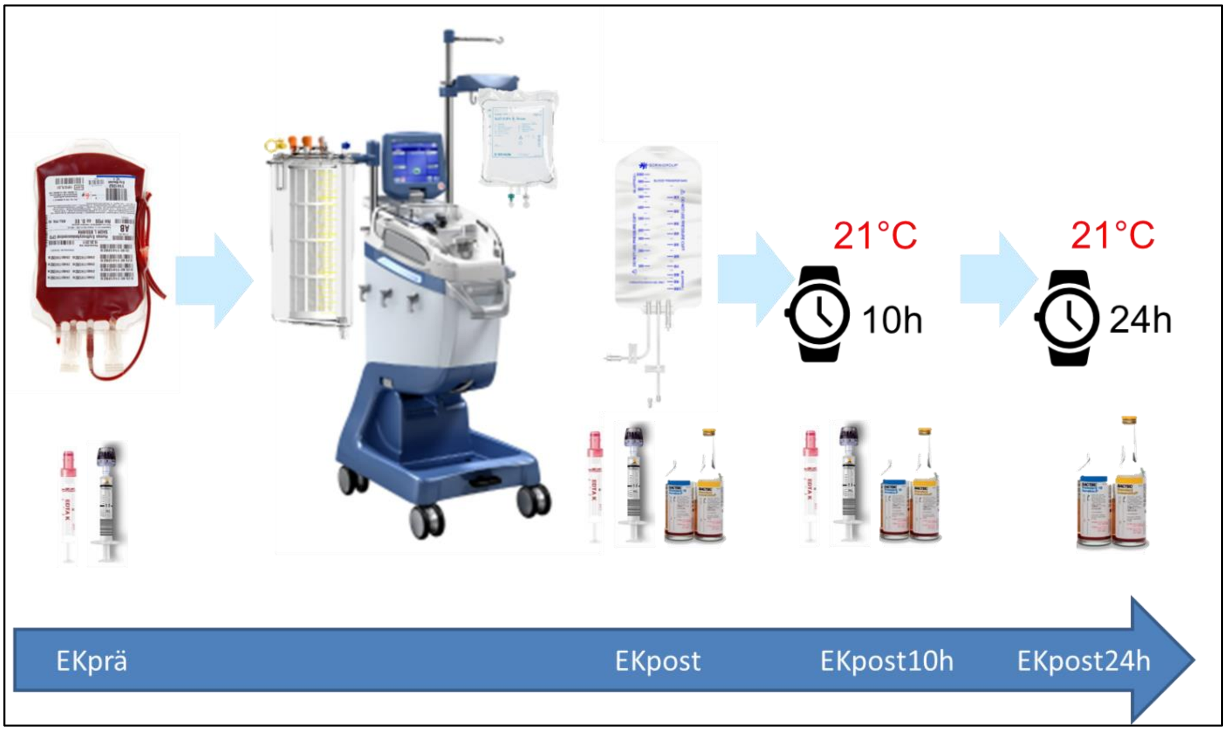

Abb. 1 ▲ *Schematische Darstellung des Versuchsablaufs mit Messpunkten: EKprä (vor MAT-Waschung, EKpost (nach MAT-Waschung, EKpost10h (10 h Lagerung im Retransfusionsbeutel bei Raumtemperatur) und EKpost24h (24 h Lagerung im Retransfusionsbeutel bei Raumtemperatur)*

Das MAT-Gerät (Xtra; LivaNova) wurde mit einer 175 ml Latham-Glocke (Bowl Set X/175, LivaNova) bestückt und mit einem Reservoir (Xres T Blood Collection Reservoir Filtered 40 µm Top Outlet, LivaNova) verbunden. An die Zufuhr „Waschlösung" zur MAT-Glocke wurde gruppenabhängig entweder NaCl 0,9 % 3.000 ml (Fresenius, Fresenius Kabi AG) oder eine handelsübliche Hämofiltrationslösung mit 4 mmol/l Kalium (Duosol 5.000 ml, B. Braun) angeschlossen. Über ein Infusionssystem (Intrafix, Braun) wurde das Reservoir mit 1000 ml Jonosteril Vollelektrolyt-Infusionsllösung (Fresenius Kabi) und 25.000 I.E. Heparin (Heparin-Natrium-25.000-ratiopharm, Ratiopharm) vorgefüllt. Ein EK wurde unter sterilen Kautelen mit einem Transfusionsbesteck (Sangofix®, B.Braun), unter Umgehung des Tiefenfilters, mit 150 mmHg Vakuum in das Reservoir gesaugt. Mittels 3-Wege-Hahn vor dem Reservoir wurde eine Probe (EKprä) entnommen und in sterile Auffangbehältern (Sarstedt) zur Weiterverarbeitung überführt.

Das MAT-Gerät wird mit dem standardisierten Waschprogramm „Popt" betrieben. Vor dem MAT-Waschen wurde das Reservoir händisch geschwenkt und anschließend die Zentrifuge mit 5.600 U/min gestartet. Initial wurde die Glocke mit 300 ml/min Reservoir-Blut gefüllt. Im Anschluss wurden nach Studienprotokoll 1.000 ml NaCl 0,9 % (Gruppe N) bzw. Hämofiltrationslösung (Gruppe HF) mit einer

Geschwindigkeit von 450 ml/min zum Waschen zugeführt. Geleert wurde die Zentrifuge mit 400 ml/min über die „Leeren“-Linie, an deren Ende sich ein angeschlossener Retransfusionsbeutel befindet. Nach der ersten aufbereiteten Glocke reichte das verbleibende Volumen im Reservoir nicht aus, um eine zweite komplette Glocke aus dem Reservoir zu bedienen. Aus diesem Grund wurde das Programm „letzte Glocke“ gewählt. Hierbei kann die Waschglocke nur zum Teil aus dem Reservoir gefüllt werden. Um das weiter benötigte Volumen zu generieren, wird schon aufbereitetes Volumen aus dem Retransfusionsbeutel rückgeführt. Nach Abschluss des Zyklus „letzte Glocke“ wurde das aufbereitete Produkt beprobt (EKpost).

Der Retransfusionsbeutel wurde vom MAT-Gerät diskonnektiert und die Abnahmestelle mit sterilem Kombistopfen verschlossen. Bis zur Messung 10 h (EKpost10h) bei Raumtemperatur wurde das gewaschene EK gelagert. Nach dieser Zeit erfolgte eine leichte Durchmischung der Lösung mit der anschließenden Abnahme der Proben **(**Fehler! Verweisquelle konnte nicht gefunden werden.**).**

Zur Bestimmung des ATP-Gehalts wurden die Blutproben nach der Abnahme direkt weiterverarbeitet. Für die ATP-Bestimmung wurden aus einem Falcon 1 ml Blut in ein 11,5 ml Glasröhrchen (Sarstedt) auf Eis pipettiert, welches 1 ml 12 %ige Trichloressigsäure (Rolf Greiner BioChemica) enthielt. Die Probe wurde mithilfe eines Vortex-Geräts gemischt und für 5 min bei 4°C gelagert. Anschließend erfolgte die Zentrifugation bei 22°C, 4.350 RPM, 10 min (Rotanta 460R, Hettich AG). Der proteinfreie Überstand wurde abpipettiert, in CryoTubes (Thermo Fisher Scientific) überführt und anschließend bei -80°C gelagert und später en bloc ausgewertet. Hierfür wurde ein Kit für die quantitative In-vitro-Bestimmung von ATP in Blut und EK mit der Hexokinase-Methode an photometrischen Systemen (Kit-Nr.1 6201, DiaSys GmbH) verwendet. Die untere Nachweisgrenze beträgt 1,5 µmol/dl. Der Referenzbereich ist im Blut definiert von 38 – 62 µmol/dl und bei EK zwischen 3,65 und 4,45 µmol/gHb. Die Plasma-Proben wurden mittels einem automatisierten Analysegerät (Cobas 6000, Roche) ausgewertet; neben den Elektrolyten (Na, K, Cl und Ca) erfolgte die Konzentrationsbestimmung der beiden Metabolite Laktat und Glukose.
